# Interobserver agreement of transvaginal ultrasound and magnetic resonance imaging in local staging of cervical cancer

**DOI:** 10.1002/uog.23662

**Published:** 2021-11-01

**Authors:** K. Pálsdóttir, S. Fridsten, L. Blomqvist, Z. Alagic, D. Fischerova, A. Gaurilcikas, K. Hasselrot, F. Jäderling, A. C. Testa, A. Sundin, E. Epstein

**Affiliations:** ^1^ Department of Women's and Children's Health Karolinska Institutet Stockholm Sweden; ^2^ Division of Pelvic Cancer, Theme Cancer Karolinska University Hospital Stockholm Sweden; ^3^ Department of Molecular Medicine and Surgery Karolinska Institutet Stockholm Sweden; ^4^ Department of Diagnostic Radiology Karolinska University Hospital Stockholm Sweden; ^5^ Department of Clinical Science Intervention and Technology, Karolinska Institutet Stockholm Sweden; ^6^ Gynecologic Oncology Center, Department of Obstetrics and Gynecology, First Faculty of Medicine Charles University and General University Hospital in Prague Prague Czech Republic; ^7^ Obstetrics and Gynecology Lithuanian University of Health Sciences Kaunas Lithuania; ^8^ Department of Obstetrics and Gynecology Danderyd Hospital Stockholm Sweden; ^9^ Department of Clinical Sciences, Danderyd Hospital Division of Obstetrics and Gynecology, Karolinska Institutet Stockholm Sweden; ^10^ Department of Radiology Capio S:t Göran Hospital Stockholm Sweden; ^11^ Dipartimento Scienze della Salute della Donna e del Bambino Fondazione Policlinico Universitario A. Gemelli, IRCCS Rome Italy; ^12^ Dipartimento Scienze della Vita e Sanità Pubblica Università Cattolica del Sacro Cuore Rome Italy; ^13^ Department of Surgical Sciences, Section for Radiology, Uppsala University Uppsala University Hospital Uppsala Sweden; ^14^ Department of Clinical Science and Education, Karolinska Institutet and Department of Obstetrics and Gynecology Södersjukhuset Stockholm Sweden

**Keywords:** MRI, neoplasm staging, observer variation, ultrasonography, uterine cervical neoplasm

## Abstract

**Objective:**

To evaluate interobserver agreement for the assessment of local tumor extension in women with cervical cancer, among experienced and less experienced observers, using transvaginal ultrasound (TVS) and magnetic resonance imaging (MRI).

**Methods:**

The TVS observers were all gynecologists and consultant ultrasound specialists, six with and seven without previous experience in cervical cancer imaging. The MRI observers were five radiologists experienced in pelvic MRI and four less experienced radiology residents without previous experience in MRI of the pelvis. The less experienced TVS observers and all MRI observers underwent a short basic training session in the assessment of cervical tumor extension, while the experienced TVS observers received only a written directive. All observers were assigned the same images from cervical cancer patients at all stages (*n* = 60) and performed offline evaluation to answer the following three questions: (1) Is there a visible primary tumor? (2) Does the tumor infiltrate > ⅓ of the cervical stroma? and (3) Is there parametrial invasion? Interobserver agreement within the four groups of observers was assessed using Fleiss kappa (κ) with 95% CI.

**Results:**

Experienced and less experienced TVS observers, respectively, had moderate interobserver agreement with respect to tumor detection (κ (95% CI), 0.46 (0.40–0.53) and 0.46 (0.41–0.52)), stromal invasion > ⅓ (κ (95% CI), 0.45 (0.38–0.51) and 0.53 (0.40–0.58)) and parametrial invasion (κ (95% CI), 0.57 (0.51–0.64) and 0.44 (0.39–0.50)). Experienced MRI observers had good interobserver agreement with respect to tumor detection (κ (95% CI), 0.70 (0.62–0.78)), while less experienced MRI observers had moderate agreement (κ (95% CI), 0.51 (0.41–0.62)), and both experienced and less experienced MRI observers, respectively, had good interobserver agreement regarding stromal invasion (κ (95% CI), 0.80 (0.72–0.88) and 0.71 (0.61–0.81)) and parametrial invasion (κ (95% CI), 0.69 (0.61–0.77) and 0.71 (0.61–0.81)).

**Conclusions:**

We found interobserver agreement for the assessment of local tumor extension in patients with cervical cancer to be moderate for TVS and moderate‐to‐good for MRI. The level of interobserver agreement was associated with experience among TVS observers only for parametrial invasion. © 2021 The Authors. Ultrasound in Obstetrics & Gynecology published by John Wiley & Sons Ltd on behalf of International Society of Ultrasound in Obstetrics and Gynecology.

**CONTRIBUTION**

**What are the novel findings of this work?**

After a short basic training session, there was similar interobserver agreement for assessment of local tumor extension in women with cervical cancer in a group of less experienced observers compared with a group of more experienced observers, both for transvaginal ultrasound (TVS) and for magnetic resonance imaging (MRI), with the exception of assessment of parametrial invasion by TVS. There was generally better agreement among MRI than among TVS observers.

**What are the clinical implications of this work?**

MRI is less dependent on observer experience than is TVS in evaluating women with cervical cancer, although interobserver agreement can be improved with a short basic training session.

## Introduction

The International Federation of Gynecology and Obstetrics (FIGO) staging system for cervical cancer was revised in 2018 to allow imaging findings, if available, to be used to assign stage, thus enabling a more accurate evaluation of local and systemic spread^1^. This was an important milestone for management and therapeutic decision‐making in these patients, addressing the discrepancies between FIGO 2009 clinical staging and surgical results, these being mainly clinical overestimation of tumor size, failure to detect parametrial invasion and failure to assess lymph‐node status^2^, ^3^. Furthermore, the interobserver agreement for clinical staging is, at best, moderate^4^.

Accepted imaging modalities for FIGO staging of cervical cancer are magnetic resonance imaging (MRI), computed tomography (CT), positron emission tomography/CT (PET/CT) and transvaginal ultrasound (TVS)/transrectal ultrasound, the method of choice depending on local availability and expertise. MRI is widely accepted for cervical cancer imaging, having high accuracy for determination of tumor size (especially lesions > 10 mm in longest diameter) and high specificity (> 90%) in the detection of parametrial invasion. MRI also allows evaluation of tumor extension in relation to adjacent organs, such as the urinary bladder and rectum, and evaluation of pelvic lymph‐node status^5^, ^6^, ^7^, ^8^. TVS and transrectal ultrasound both have high accuracy (> 90%) for tumor detection and high specificity for identification of parametrial invasion. TVS is especially valuable for small tumors ≤ 1 cm in longest diameter^9^, ^10^, ^11^, ^12^. An important aspect of TVS is that image acquisition and interpretation are usually performed during the examination and are thus dependent on the operator's technical skills and expertise. In contrast, MRI acquisition is performed according to routine protocols in standardized anatomical planes, with evaluation being carried out after the examination. The operator dependency of TVS has led to doubts regarding its reproducibility for evaluation of cervical cancer. A few studies have been published on the interobserver agreement between experienced MRI observers^13^, ^14^, but no study has addressed how ultrasound or MRI observer experience affects the evaluation of patients with cervical cancer.

The aim, therefore, of this study was to assess, in a cohort of cervical‐cancer patients, the interobserver agreement among experienced and less experienced observers using TVS and MRI.

## Subjects and methods

This study was performed at Karolinska University Hospital in Stockholm, Sweden and was approved by the regional ethics committee (Dnr 2011/1925‐31/3). Written informed consent to participate was obtained from all patients.

### Study participants

During the study period, between July 2011 and August 2015, all women with invasive cervical cancer verified by biopsy in the region of Stockholm/Gotland and referred to the tertiary‐level Karolinska University Hospital, in Stockholm, Sweden, were eligible for inclusion in this study, irrespective of disease stage. Before referral, some of the women had undergone a diagnostic cone biopsy, not all of them had residual disease according to findings on radical surgery. Exclusion criteria included: pregnancy at the time of diagnosis, histological diagnosis other than cervical cancer verified after surgery and insufficient imaging data. TVS data were considered insufficient when there were only still images, an absence of Doppler images or cine‐clips or cine‐clips not including the entire parametrium. MRI data were considered insufficient when there was a lack of pulse sequences, such as contrast‐enhanced images, or there were artifacts from metal implants, severe motion‐related artifacts or severe obesity impairing image quality.

Following referral, all women underwent a routine work‐up, including MRI and clinical examination under anesthesia for clinical staging according to FIGO's revised 2009 staging criteria^15^. Women with early‐stage disease (≤ FIGO stage IB1) underwent additional radical surgical treatment, while those with advanced‐stage disease received radiochemotherapy. TVS was also performed as part of the study protocol. Baseline demographic data, including age, histology type, prior diagnostic cone biopsy and clinical stage, were collected prospectively. Information on dates of MRI and TVS examinations and surgery for women with early‐stage disease was also collected.

### Transvaginal ultrasound

All TVS examinations were performed by one of two ultrasound experts, one with 17 years' (E.E.) and one with 3 years' (K.P.) experience as a consultant sonographer, using a Voluson E8 ultrasound system (GE Healthcare, Zipf, Austria), equipped with a 5–9‐MHz three‐dimensional transvaginal transducer, or an IU22 ultrasound system (Philips Healthcare, Best, The Netherlands), equipped with a 3–9‐MHz transvaginal transducer. The examination was performed transvaginally, applying a standardized protocol according to Fischerova's systematic method^16^, with the woman in the lithotomy position, having emptied her bladder prior to the examination. Still images and cine‐clips of conventional grayscale and power Doppler ultrasound examinations were recorded in sagittal and transverse planes, with the image optimized focusing on the uterine cervix. The image set recorded for each patient included between three and 10 cine‐clips, with and without Doppler. When Doppler cine‐clips were missing, still Doppler images of the cervix were instead provided in the image set. All ultrasound images were free from any annotations indicating findings. Images and cine‐clips were coded, assigned a number randomly and stored on flash drives. The ultrasound examiner who prepared the ultrasound image material for the observers and gave a short training workshop (K.P.) did not participate in the interobserver evaluation.

### MRI

MRI was performed using one of four different MRI scanners (1.5‐Tesla (T) Magnetom Aera, 1.5‐T Magnetom Avanto or 3‐T Magnetom Verio (Siemens Healthineers AG, Erlangen, Germany) or 1.5‐T Intera (Philips Medical Systems)) using a phased‐array body coil. To minimize bowel motion‐related artifacts, the patient fasted for at least 4 h prior to imaging and received an intramuscular injection of antiperistaltic agent, either 1‐mg glucagon (Glucagen® (Novo Nordisk, Bagsværd, Denmark)) or 20‐mg butyl‐scopolamine (Buscopan® (Boehringer Ingelheim GmbH, Ingelheim, Germany)). They were also instructed to use a small enema to empty the rectum before arriving at the radiology department. Acquisition included high‐resolution T2‐weighted axial and sagittal images, oblique coronal images (i.e. along the longitudinal axis of the tumor/cervical canal) and oblique transaxial images (i.e. perpendicular to the tumor/cervical canal) as well as T1‐weighted transaxial/transverse images of the pelvis before and after intravenous administration of a gadolinium‐chelate‐based contrast agent (Gadopentetic acid, Magnevist®, 469 mg/mL, 0.2 mL/kg body weight (Bayer AB, Solna, Sweden) or Gadoteric acid, Dotarem®, 279.3 mg/mL, 0.2 mL/kg body weight (Gothia Medical AB, Billdal, Sweden)). Diffusion‐weighted imaging (DWI) was not performed for all patients and hence was not reviewed for the purposes of this study. All MR images were free from any annotations indicating findings. The patients received the same study number for MRI as they had been allocated for TVS. The radiologist who prepared the MR images for the observers and held the short training workshop (S.F.) did not participate in the interobserver evaluations.

### Image review and observer performance

For both TVS and MRI, there were two groups of observers: a group of experts in cervical cancer imaging and a group without such previous experience (Table S1). The experienced TVS observers were six European gynecologists and consultant ultrasound specialists with 3–18 years of experience in cervical cancer imaging (‘experienced’ group). The TVS observers with less experience were seven Swedish gynecologists and consultant ultrasound specialists with 1–2 years of experience in gynecological ultrasound, but not cervical cancer imaging (‘less experienced’ group). The MRI observers were five specialists in radiology with 5–25 years of experience in abdominal radiology, including 1–25 years of experience in MRI of the pelvis (‘experienced’ group), and four radiology residents with 3–5 years of experience in general radiology but without previous experience in MRI of the pelvis (‘less experienced’ group).

All TVS observers received a written manual on how to review the TVS examinations. The less experienced observers additionally attended a 1‐h lecture/workshop by an ultrasound expert (K.P.) on TVS imaging of cervical cancer, with emphasis on tumor assessment and detection of stromal and parametrial invasion, according to Fischerova's method^16^. Using their own personal computer with a high‐resolution screen, the observers then performed individually an offline evaluation of the coded TVS image/cine‐clip sets, which were provided to them on the flash drives. The observers were blinded to all imaging results and the patients' demographic and clinical information, except for their cervical cancer diagnosis.

All MRI observers were introduced to MRI of cervical cancer during a 2‐h workshop by a radiologist specializing in cervical cancer imaging (S.F.). Over the following 2 days, they reviewed the coded MRI examinations on the Karolinska University Hospital PACS system (SECTRA PACS, IDS7, version 19.3.6.3510, Linköping, Sweden).

All observers submitted their imaging findings using their own unique, anonymized link to an online survey (Survey Monkey®; see links: TVS Survey (http://www.huxa.net/kp/survey‐us.html) and MRI Survey (http://www.huxa.net/kp/survey‐mri.html). For each modality, a complete imaging assessment was submitted consecutively for each patient before continuing to the next patient, although it remained possible to edit all imaging evaluations until the survey was completed. For each case, the observers answered the following three questions: (1) Is there a visible primary tumor? (Yes/No); (2) Does the tumor infiltrate > ⅓ of the cervical stroma? (Yes/No); and (3) Is there parametrial invasion? (Yes/No). For each question, they additionally rated their confidence regarding the particular finding, on a visual analog scale (VAS) ranging between 0 and 100, where 0 = very uncertain and 100 = absolutely confident. They also rated the image quality for each examination on a VAS of 0–100, where 0 = very unsatisfactory and 100 = perfect.

### Statistical analysis

The chi‐square test was used for all categorical data, the independent two‐sample *t*‐test was used for normally distributed continuous data and the Mann–Whitney *U*‐test was used for non‐normally distributed data. Fleiss kappa (κ) was calculated for each of the groups of experienced and less experienced TVS and MRI observers. Agreement was interpreted as being: poor for κ = 0–0.2, fair for κ = 0.21–0.40, moderate for κ = 0.41–0.60, good for κ = 0.61–0.80 and very good for κ = 0.81–1^17^. Observer confidence and image quality (VAS) was tested with the Wilcoxon rank‐sum test, and Spearman's correlation was performed to correlate observer confidence with image quality. For women who underwent surgery, histology was used as the gold standard to calculate sensitivity and specificity. The statistical analysis was performed using SPSS (version 25, Statistical Package for Social Science, IBM Corp., Armonk, NY, USA). The level of significance was set at *P* < 0.05. Data are given as mean or median, depending on the distribution, with 95% CI.

## Results

### Patients

During the study period, from July 2011 to August 2015, 483 patients were diagnosed with cervical cancer and referred to our hospital. Of these, 89 agreed to participate and were eligible for inclusion in the present study, of whom we excluded 29 for the following reasons: cancer in the cervical stump (*n* = 1), cancer *in situ* (*n* = 1), lack of MRI data (*n* = 10), insufficient TVS and MRI data (*n* = 3), insufficient MRI data (*n* = 3) and insufficient TVS data (*n* = 11). Thus, the final study group comprised 60 patients. Their demographic data are given in Table 1. The median time interval between TVS and MRI examinations was 5 (range, 0–45) days, that from MRI to radical surgery was 42 (range, 4–71) days and that from TVS to radical surgery was 36 (range, 9–69) days.

**Table 1:** Demographic characteristics of the study group of 60 patients with cervical cancer

Characteristic	Value
Age (years)	46 (24–85)
Tumor stage (FIGO)	
IA1	1 (1.7)
IA2	1 (1.7)
IB1	34 (56.7)
IB2	1 (1.7)
IIA	7 (11.7)
IIB	13 (21.7)
IIIB	2 (3.3)
IV	1 (1.7)
Histology	
Squamous cell carcinoma	40 (66.7)
Adenocarcinoma	18 (30.0)
Other	2 (3.3)
Treatment	
Diagnostic cone biopsy	19 (31.7)
Surgery	31 (51.7)
Radiochemotherapy	29 (48.3)

Data are given as median (range) or *n* (%).

FIGO, International Federation of Gynecology and Obstetrics.

### Interobserver agreement

In Table 2, we present the interobserver agreement (κ‐values) within the observer groups. Experienced and less experienced TVS observers showed moderate interobserver agreement, ranging from 0.44 to 0.57 for tumor detection, stromal invasion and parametrial invasion. Regarding parametrial invasion, the experienced TVS observers showed significantly better agreement than did the less experienced ones, with κ‐value of 0.57 (95% CI, 0.51–0.64) *vs* 0.44 (95% CI, 0.39–0.50). Both experienced and less experienced MRI observers achieved good interobserver agreement, with κ‐values of 0.69–0.80 for stromal invasion and parametrial invasion, while, for tumor detection, the agreement was moderate (κ, 0.51 (95% CI, 0.41–0.62)) for the less experienced observers and good for the experienced ones (κ, 0.70 (95% CI, 0.62–0.78)). In a subanalysis of 41 patients, excluding the 19 patients who had undergone cone biopsy prior to imaging, κ‐value was unchanged for all TVS observers (data not shown), but was lower, although not statistically significantly so, for the combined group of experienced and less experienced MRI observers with respect to tumor detection, changing from 0.64 (95% CI, 0.60–0.68) to 0.56 (95% CI, 0.51–0.61).

**Table 2:** Interobserver agreement for groups of experienced and less experienced transvaginal ultrasound (TVS) and magnetic resonance imaging (MRI) observers interpreting imaging datasets from patients with cervical cancer

Observers	Fleiss *κ*	95% CI	Agreement
(1) Tumor seen (yes/no)			
TVS			
Experienced (*n* = 6)	0.46	0.40–0.53	Moderate
Less experienced (*n* = 7)	0.46	0.41–0.52	Moderate
Whole group (*n* = 13)	0.46	0.43–0.49	Moderate
MRI			
Experienced (*n* = 5)	0.70	0.62–0.78	Good
Less experienced (*n* = 4)	0.51	0.41–0.62	Moderate
Whole group (*n* = 9)	0.64	0.60–0.68	Good
(2) Cervical stromal invasion >⅓ (yes/no)			
TVS			
Experienced (*n* = 6)	0.45	0.38–0.51	Moderate
Less experienced (*n* = 7)	0.53	0.40–0.58	Moderate
Whole group (*n* = 13)	0.50	0.47–0.53	Moderate
MRI			
Experienced (*n* = 5)	0.80	0.72–0.88	Good
Less experienced (*n* = 4)	0.71	0.61–0.81	Good
Whole group (*n* = 9)	0.73	0.69–0.77	Good
(3) Parametrial invasion (yes/no)			
TVS			
Experienced (*n* = 6)	0.57	0.51–0.64	Moderate
Less experienced (*n* = 7)	0.44	0.39–0.50	Moderate
Whole group (*n* = 13)	0.51	0.48–0.54	Moderate
MRI			
Experienced (*n* = 5)	0.69	0.61–0.77	Good
Less experienced (*n* = 4)	0.71	0.61–0.81	Good
Whole group (*n* = 9)	0.68	0.64–0.72	Good

Agreement was interpreted as: poor for κ = 0–0.2, fair for κ = 0.21–0.40, moderate for κ = 0.41–0.60, good for κ = 0.61–0.80 and very good for κ = 0.81–1^17^.

### Observer confidence and assessment of image quality

In Table 3, we present the VAS scores for observer confidence and assessment of image quality for the different observer groups. Experienced TVS observers were more confident than were less experienced TVS observers for all three components of the assessment. There was a positive correlation between observer confidence and their assessment of image quality for the experienced TVS observers regarding all three components of the assessment, with the strongest correlation being for tumor detection (ρ = 0.672, *P* < 0.001). There was likewise a positive correlation between observer confidence and image quality for the less experienced TVS observers regarding tumor detection and >⅓ stromal invasion but not for parametrial invasion (ρ = 0.214, *P =* 0.1).

**Table 3:** Observer confidence regarding imaging findings and assessment of image quality, when reviewing transvaginal ultrasound (TVS) and magnetic resonance imaging (MRI) datasets from patients with cervical cancer

Observers	Observer confidence (VAS score)	Image quality (VAS score)	Spearman's (ρ)	*P* *
(1) Tumor seen				
TVS				
Experienced (*n* = 6)	100 (93–100)	80 (73–83)	0.672	< 0.001
Less experienced (*n* = 7)	80 (71–85)	76 (71–80)	0.573	< 0.001
*P* †	< 0.001			
MRI				
Experienced (*n* = 5)	100 (99–100)	85 (84–89)	0.153	0.243
Less experienced (*n* = 4)	92 (84–98)	71 (68–73)	0.318	0.010
*P* †	< 0.001			
(2) Cervical stromal invasion >⅓				
TVS				
Experienced (*n* = 6)	90 (80–95)	80 (73–83)	0.468	< 0.001
Less experienced (*n* = 7)	74 (70–80)	76 (71–80)	0.463	< 0.001
*P* †	< 0.001			
MRI				
Experienced (*n* = 5)	100 (100–100)	85 (84–89)	0.221	0.090
Less experienced (*n* = 4)	90 (87–95)	71 (68–73)	0.220	0.090
*P* †	< 0.001			
(3) Parametrial invasion				
TVS				
Experienced (*n* = 6)	85 (80–91)	80 (73–83)	0.297	0.020
Less experienced (*n* = 7)	73 (70–77)	76 (71–80)	0.214	0.100
*P* †	< 0.001			
MRI				
Experienced (*n* = 5)	99 (90–100)	85 (84–89)	0.166	0.204
Less experienced (*n* = 4)	82 (78–90)	71 (68–73)	0.090	0.488
*P* †	< 0.001			

Observer confidence and image quality presented as median (95% CI) visual analog scale (VAS) scores.

* Wilcoxon rank‐sum *P* < 0.05 is significant for Spearman's correlation between medians.

† Wilcoxon rank‐sum for experienced *vs* less experienced observers.

For all parts of the MRI assessment, the experienced MRI observers were more confident than were the less experienced observers (*P* < 0.001). Observer confidence and assessment of image quality were unrelated, except among the less experienced observers for tumor detection (ρ = 0.318, *P* = 0.01).

Experienced MRI observers rated image quality as being higher than did experienced TVS observers, with median (95% CI) VAS scores of 85 (84–89) and 80 (73–83), respectively (*P* = 0.004). The reverse was true for the less experienced observers, with the less experienced TVS observers rating image quality as being higher compared with the less experienced MRI observers, with median (95% CI) VAS scores of 76 (71–80) and 71 (68–73), respectively (*P* < 0.001). Table S2 gives the sensitivities and specificities for tumor detection and cervical stromal invasion on TVS and MRI assessment in the 31 women who underwent radical surgery.

## Discussion

In this study, we found the interobserver agreement for assessment of primary tumor extension in patients with cervical cancer to be moderate for TVS and moderate‐to‐good for MRI. An unexpected finding was the similarity in interobserver agreement (according to Fleiss κ) of experienced and less experienced observers, both for TVS and for MRI. Only for parametrial invasion was there a difference, with experienced TVS observers showing significantly better agreement than did less experienced observers. Since this study was not designed as a diagnostic accuracy study, the sensitivities and specificities for the observer groups are included only for reference in Table S2. There were no significant differences between experienced and less experienced observers in sensitivity or specificity with respect to tumor detection or stromal invasion, for either TVS or MRI, further supporting our findings regarding the similarity in interobserver agreement.

To our knowledge, there have been no results on interobserver variability for ultrasound in women with cervical cancer published previously. However, in patients with endometrial cancer, Eriksson and colleagues reported similar agreement between ultrasound observers, irrespective of their previous experience, for the assessment of myometrial invasion, although, for the assessment of cervical stromal invasion, they found that experienced TVS observers had better agreement than did less experienced observers (all gynecologists)^18^. The basic training together with the repetition of the image review process in both their study and ours might have influenced the agreement and clinical performance of the less experienced groups. Previous studies have reported on the reproducibility of MRI and CT in cervical cancer. Our results show considerably higher agreement for the MRI observers than in a previous retrospective study of 152 patients with early‐stage disease examined by experienced observers (κ = 0.32 for tumor detection and 0.11 for parametrial invasion)^19^. However, that study included patients with less advanced tumors than those included in our study population, so the results are not directly comparable, as tumor invasion is easier to identify in more advanced‐stage disease. Also, we used a dichotomized scoring system, as opposed to the multiple choice questions of Hricak *et al*.^19^, which allowed for greater variation in the evaluation and consequently for greater interobserver variability in their study. Furthermore, MRI quality has improved since their study, published in 2007, was performed.

At our institution, experienced MRI observers do not perform assessment of cervical stromal invasion routinely. The clinical importance of stromal invasion is questionable, although, since the Gynecologic Oncology Group Study #92, published in 1999, it has been used, together with tumor size and lymph vascular space invasion, to select patients for adjuvant treatmen postoperatively^20^.

Before imaging, a significant proportion (32% (19/60)) of our patients had undergone diagnostic cone biopsy. This reflects the typical clinical situation, in which approximately 30% of all cervical cancers are found by screening, especially in younger patients^21^, ^22^. Previous studies have shown that edema and hemorrhage following cone biopsy may affect the interpretation of MRI^23^, ^24^ and can mimic small tumors, while this does not appear to be the case for Doppler ultrasound^10^. This may have resulted in more false‐positive cases among conized patients on MRI, leading to a drop in κ‐values for the MRI observers when these patients were excluded, although the difference was not statistically significant.

Our findings should be interpreted in light of the study set‐up, with very limited clinical information being made available to the observers and the evaluation protocol allowing only a dichotomized assessment of the imaging findings. The intrinsic differences between the setting of the experimental offline TVS assessment and the unique, dynamic TVS examination technique in the clinical setting may also have impacted on the results. For instance, the possibility to sense the firmness of a cervical tumor during a dynamic examination is not experienced in the offline setting and this could have affected the assessment, especially with respect to parametrial invasion. Furthermore, the larger size of the observer group for TVS than for MRI may have affected their relative interobserver variabilities, while the fact that not all of the TVS observers, only the less experienced ones, attended the initial teaching session could have affected the TVS results. Additionally, the fact that the TVS datasets were assessed by experts at multiple institutions, rather than at a single center, as was the case for the MRI assessment, may also have affected the results, due to probable differences in the frame of reference for imaging evaluations at different centers, affecting generalizability. It is interesting that there was a positive correlation between image quality and observer confidence for TVS but not for MRI observers, indicating that the former may have been more dependent on image quality in the offline setting to feel confident in their assessment. It is a potential weakness of the study that, due to a change in MRI protocols during the study period, only the basic common sequences could be used in our analysis, excluding DWI, as DWI may improve tumor detection and the evaluation of local tumor spread^25^, ^26^. Strengths of this study are the mixed cohort of patients, including all stages of cervical cancer, and that the imaging assessment was performed by four different observer groups, each including multiple observers with various levels of experience. Furthermore, the study data were complete for all observers because of the mandatory questions in the Survey Monkey questionnaire.

In conclusion, we found that interobserver agreement for the assessment of local tumor extension in patients with cervical cancer was moderate for TVS and moderate‐to‐good for MRI. The level of interobserver agreement was associated with observer experience only among TVS observers for parametrial invasion. Our results indicate that, with a short basic training session, acceptable interobserver agreement is achieved in experienced and less experienced groups of observers in the evaluation of cervical cancer imaging with both TVS and MRI.

## Supporting information


**Table S1** Experience of the observers participating in the study
**Table S2** Sensitivity and specificity for tumor detection and cervical stromal invasion on transvaginal ultrasound (US) and magnetic resonance imaging (MRI) of all observer groups for patients with early‐stage cervical cancer (*n* = 31)

## Data Availability

Data available on request only due to privacy/ethical restrictions
