# Supplementary material for: Interobserver agreement of transvaginal ultrasound and magnetic resonance imaging in local staging of cervical cancer
Source: Ultrasound Obstet Gynecol. 2021 Nov 1;58(5):773–9. doi: 10.1002/uog.23662 (PMC8597592; doi:10.1002/uog.23662)
Supplement: Supplementary file 1 — Table S1 Experience of the observers participating in the study Table S2 Sensitivity and specificity for tumor detection and cervical stromal invasion on transvaginal ultrasound (US) and magnetic resonance imaging (MRI) of all observer groups for patients with early‐stage cervical cancer (n = 31) [file UOG-58-773-s001.docx]

**Table S1** Experience of the observers participating in the study

| *Observers* |  |  |  |
| --- | --- | --- | --- |
| *US Experienced* | *Ob/Gyn specialist* | *Second opinion US* | *CC assessment US* |
| Observer 1 | 24 | 18 | 18 |
| Observer 2 | 15 | 13 | 13 |
| Observer 3 | 23 | 15 | 9 |
| Observer 4 | 11 | 7 | 11 |
| Observer 5 | 5 | 3 | 3 |
| Observer 6 | 10 | 7 | 10 |
| Median (IQR) | 13 (15) | 10 (10) | 10.5 (7) |
| *US Less experienced* |  |  |  |
| Observer 1 | 4 | 2 | 0 |
| Observer 2 | 4 | 2 | 0 |
| Observer 3 | 2 | 1 | 0 |
| Observer 4 | 5 | 1 | 0 |
| Observer 5 | 8 | 1 | 0 |
| Observer 6 | 5 | 1 | 0 |
| Observer 7 | 5 | 1 | 0 |
| Median (IQR) | 5 (1) | 1 (1) | 0 |
| *MRI Experienced* | *Radiology experience* | *MRI experience* | *Pelvic MRI experience* |
| Observer 1 | 10.5 | 4.5 | 1.5 |
| Observer 2 | 20 | 15 | 10 |
| Observer 3 | 30 | 25 | 20 |
| Observer 4 | 17 | 8 | 8 |
| Observer 5 | 18 | 13 | 13 |
| Median (IQR) | 18 (11.3) | 13 (13.8) | 10 (11.8) |
| *MRI Less experienced* |  |  |  |
| Observer 1 | 3 | 0 | 0 |
| Observer 2 | 4 | 0 | 0 |
| Observer 3 | 4 | 0 | 0 |
| Observer 4 | 5 | 0 | 0 |
| Median (IQR) | 4 (1.5) | 0 | 0 |

Data are given as years. Ob/Gyn = obstetrics and gynecology, US = ultrasound,

CC = cervical cancer, IQR = interquartile range, MRI = magnetic resonance imaging

**Table S2** Sensitivity and specificity of all observer groups for patients with early-stage disease (*n* = 31)

*Tumor detection* *Stromal invasion >⅓*

*Observers Sensitivity Specificity Sensitivity Specificity*

MRI experienced 68 (55–81) 85 (78–92) 60 (51–69) 93 (93)

MRI less experienced 72 (57–88) 69 (42–100) 72 (60–84) 82 (64–100)

*P 0.46 (ns) 0.90 (ns) 0.06 (ns) 0.06 (ns)*

US experienced 85 (77–93) 36 (16–55) 65 (51–79) 74 (50–97)

US less experienced 79 (71–86) 52 (44–60) 48 (31–66) 86 (77–94)

*P 0.18 (ns) 0.10 (ns) 0.10 (ns) 0.30 (ns)*

Sensitivity (%) and specificity (%) are presented as mean (95% CI). Histology from surgical specimen. ns, not significant.
